# Mid-State Kalman Filter for Nonlinear Problems

**DOI:** 10.3390/s22041302

**Published:** 2022-02-09

**Authors:** Zhengwei Liu, Ying Chen, Yaobing Lu

**Affiliations:** Beijing Institute of Radio Measurement, Beijing 100854, China; lk324516@163.com (Z.L.); luyaobing65@163.com (Y.L.)

**Keywords:** consistency, Kalman filter, nonlinear systems, radar target tracking

## Abstract

When tracking very long-range targets, wide-band radars capable of measuring targets with high precision at ranges have severe measurement nonlinearities. The existing nonlinear filtering technology, such as the extended Kalman filter and untracked Kalman filter, will have significant consistency problems and loss in tracking accuracy. A novel mid-state Kalman filter is proposed to avoid loss and preserve the filtering consistency. The observed state and its first-order state derivative are selected as the mid-state vector. The update process is transformed into the measurement space to ensure the Gaussian measurement distribution and the linearization of the measurement equation. In order to verify the filter performance in comparison, an iterative formulation of Cramér-Rao Low Bound for the nonlinear system is further derived and given in this paper. Simulation results show that the proposed method has excellent performance of high filtering accuracy and fast convergence by comparing the filter state estimation accuracy and consistency.

## 1. Introduction

Target tracking is a process that uses sensors to estimate the characteristics of a moving object of interest. It is widely used in unmanned aerial vehicles (UAV), military strikes, and other fields [1,2]. As the core problem of target tracking, state estimation theory realizes the real-time online estimation of a target’s motion state by integrating the prior information of the target and online measurement information provided by sensors. When the system satisfies the linear Gaussian condition, a Kalman filter (KF) can recursively obtain the consistent minimum variance and linear unbiased estimate of the state and is the optimal solution [3]. However, the system model is nonlinear in multi-sensor fusion, radar maneuvering target tracking, and satellite communication systems [4]. For the nonlinear system model, a nonlinear filtering method is needed to improve the estimation accuracy.

The extended Kalman filter (EKF) is the most widely used nonlinear filter in practical engineering because of its simple algorithm and small computation. The EKF uses a Taylor series expansion to approximate the nonlinear system model. When the nonlinearity is severe, the filtering accuracy will be reduced or even diverged due to a high-order truncation error [5]. Therefore, a second order and higher-order EKF are proposed successively, but their computational burden increases significantly [6]. The iterated extended Kalman filter (IEKF) is also obtained by dividing the one-step update of the EKF into multiple steps in pseudo-time and gradually updating the states according to the nonlinear gradient of measurement function [7]. The scholars applied numerical integration approximation methods to nonlinear filtering. The Gauss Hermite filter (GHF), unscented Kalman filter (UKF), and cubature Kalman filter (CKF) were proposed successively. The GHF is a polynomial integral approximation filtering algorithm for nonlinear system models [8] which uses Gauss-Hermite polynomials to approximate the probability density in Gaussian filtering. The UKF takes the UT criterion to select deterministic Sigma sampling points in the original state distribution point set, inputs the sampling points into the nonlinear system, and obtains the mean and covariance of the posterior probability density function through the transformation of the point set [9,10]. The UKF has less computation and better approximation performance than the EKF. The CKF is based on the spherical-radial criterion and uses a group of cubature points with equal weight to calculate the mean and covariance of state variables [11]. Wang Shuo conducts a comparative analysis on the UKF and CKF for low-dimensional and high-dimensional models under nonlinear conditions. The simulation results show that the CKF has the optimal numerical stability and filtering accuracy under high dimensional conditions [12,13]. A Particle Filter (PF) is not limited by the linearization error or gaussian noise assumption and approximates the probability density function corresponding to the nonlinear function. However, the considerable amount of calculation is unbearable for a real-time target tracking system [14].

Due to the complexity and uncertainty of very long-range target tracking, the Gaussian noise distribution in the measurement space will become a severe non-Gaussian distribution when it is converted into the state space in the update process. This phenomenon is often encountered in wide-band radar systems with high-range accuracy [15]. Due to the nonlinear distribution of the measurement area, existing nonlinear filters have specific problems in the accuracy of state estimation and the consistency of the filter and even divergence of the filter may occur in the EKF [16]. Because of this, a mid-state Kalman filter (MSKF) is proposed in this paper. A MSKF takes the observed state and its first-order state derivative as the mid-state vector and converts the predicted state covariance into the covariance of the mid-state vector according to the third-order spherical-radial criterion. Then the KF update process is converted to the measurement space and realizes the linearization of the measurement equation. Therefore, information loss in updating will be reduced to a minimum and the MSKF will have a higher accuracy in state estimation. The MSKF was applied to very long-range tracking problems and the simulation results prove the superiority and applicability of the algorithm.

## 2. Traditional Nonlinear Filter

In practical engineering applications, discrete nonlinear systems are generally characterized using additive noise and the system model is expressed as

(1) $$\{\begin{array}{l} x_{k}=f(x_{k-1})+w_{k-1}\\ z_{k}=h(x_{k})+v_{k} \end{array}$$

where process noise $w_{k}$ and observation noise $v_{k}$ are independent white zero-mean Gaussian noise.

The EKF obtains linear approximation of nonlinear system by using Taylor series expansion and then uses a KF to deal with the filtering problem of nonlinear system. The EKF approximates the nonlinear functions $f(x_{k-1})$ and $h(x_{k})$ in the state space to the first-order Taylor polynomials near ${\hat{x}}_{k}$ and ${\hat{x}}_{k|k-1}$, respectively:

(2) $$f(x_{k})\approx f({\hat{x}}_{k-1})+F_{k-1}[x_{k-1}-{\hat{x}}_{k-1}]$$

(3) $$h(x_{k})\approx h({\hat{x}}_{k|k-1})+H_{k}[x_{k}-{\hat{x}}_{k|k-1}]$$

where $F_{k-1}$ and $H_{k}$ are the Jacobi matrices as follows:

(4) $$F_{k-1}={\frac{\partial f(x_{k-1})}{\partial x}|}_{x={\hat{x}}_{k-1}}$$

(5) $$H_{k}={\frac{\partial h(x_{k})}{\partial x}|}_{x={\hat{x}}_{k|k-1}}$$

The UKF is different from the EKF in that it directly calculates the mean and covariance of the target distribution, avoiding the approximation of nonlinear functions. The UKF uses the mean and covariance of initial distribution to generate a series of determined sigma sampling points according to Formulas (6) and (7). These sigma sampling points are propagated through nonlinear functions to get the estimated mean and covariance [17].

(6) $$\{\begin{array}{l} \chi_{0}=x_{k}\\ \chi_{i}=x_{k}+e_{i}\sqrt{(n_{x}+\kappa )P_{x}}\\ \chi_{i+n}=x_{k}-e_{i}\sqrt{(n_{x}+\kappa )P_{x}} \end{array},i=1,2,\cdots ,n_{x}$$

The corresponding weight is

(7) $$\{\begin{array}{c} w_{0}=\frac{\kappa }{n_{x}+\kappa }\\ w_{i}=w_{i+n}=\frac{1}{2(n_{x}+\kappa )} \end{array}$$

where κ is a fine-tuned scalar parameter, $n_{x}\;$ is the dimension of the state vector $x_{k}$, and $e_{i}$ is the i-th Cartesian fundamental vector. The size of κ is related to the size of the sample moments and controls the distance from the sigma point to the mean point.

Under the Gaussian assumption, the state estimation of the nonlinear KF can ultimately be equated to the calculation of multidimensional vector integral, which can be summarized in the form of a nonlinear function × Gaussian probability density function:

(8) $$I(g)=\int_{R^{n}}g(x)N(x;\bar{x},P_{x})dx$$

where g(·) is any nonlinear function. To solve the integral numerically, the CKF transforms it into spherical-radial form and then carries out numerical integration according to the spherical-radial criterion

(9) $$\int_{0}^{\infty }f(r)r^{n-1}\exp(-r^{2})dr=\sum_{i=1}^{m}w_{i}f(r_{i})$$

where r is the radial scalar obtained by spherical-radial transformation of the vector x in a cartesian coordinate system. The CKF filtering process is similar to that of the UKF. They all transform the sampled points with weight through the equations of the nonlinear system to calculate the first and second order moments required by filtering, but they have essential differences in obtaining sampling points. In a high-dimensional system, the weights of the UKF sigma points are prone to be negative which will cause the problem of unstable estimated values. The weights of the CKF sampling points are always positive, which solves the problem of instability. However, the filtering accuracy will be affected due to the increasing distance between the sampling point and the center point.

## 3. Mid-State Kalman Filter for Nonlinear Problems

The KF is proposed based on the least mean square error criterion. Under the assumption that the observation noise obeys the Gaussian distribution, the KF is a constant minimum variance and linear unbiased estimate that can recursively obtain the state. In this paper, a Mid-state Kalman Filter is proposed based on the KF method. A MSKF selects the observed state and its first-order state derivative as the mid-state vector and transforms the filter update process into the measurement space. The proposed method guarantees the Gaussian distribution of the measurement and correspondingly transforms the measurement equation into a linear form.

### Mid-State Kalman Filter

Consider a nonlinear discrete system for target tracking as shown below:

(10) $$\{\begin{array}{l} x_{k}=F_{k}x_{k-1}+w_{k-1}\\ z_{k}=H_{k}(x_{k})+v_{k} \end{array}$$

where, $x_{k}\in R^{n}$ and $z_{k}\in R^{m}$ are the system state vector and the measurement vector; $F_{k}(\cdot ):R^{n}\to R^{n}$ and $H_{k}(\cdot ):R^{n}\to R^{m}$ respectively represent the system state function and nonlinear measurement function; and the process noise $w_{k}$ and observation noise $v_{k}$ are independent zero-mean Gaussian noise with covariance $Q_{k}$ and $R_{k}$.

In the typical two-dimensional radar target tracking, the target is assumed to be in uniform linear motion. Its state vector at time k is $x_{k}={\left[x_{k},v_{x_{k}},y_{k},v_{y_{k}}\right]}^{T}$, including the position ${\left[x_{k},y_{k}\right]}^{T}$ and velocity vector ${\left[v_{x_{k}},v_{y_{k}}\right]}^{T}$. In the observation vector $z_{k}={\left[r_{k},\theta_{k}\right]}^{T}$, $r_{k}$ and $\theta_{k}$ are the distance measurement and angle measurement, respectively. The system state function and nonlinear measurement function in Equation (10) are respectively expressed as:

(11) $$F=\left[\begin{array}{cccc} 1 & T & 0 & 0\\ 0 & 1 & 0 & 0\\ 0 & 0 & 1 & T\\ 0 & 0 & 0 & 1 \end{array}\right],H_{k}(x_{k})=\left[\begin{array}{c} \sqrt{x_{k}^{2}+y_{k}^{2}}\\ \arctan(\frac{y_{k}^{2}}{x_{k}^{2}}) \end{array}\right]$$

The MSKF selects $x_{M_{k}}={\left[z_{k},{\dot{z}}_{k}\right]}^{T}={\left[r_{k},{\dot{r}}_{k},\theta_{k},{\dot{\theta }}_{k}\right]}^{T}$ as mid-state vector and linearizes the measurement equation in the measurement space as:

(12) $$z_{k}=H_{z}x_{M_{k}}+v_{k}=[\begin{array}{cc} I_{m} & 0_{m\times m} \end{array}]\times {\left[z_{k},{\dot{z}}_{k}\right]}^{T}+v_{k}$$

In this case, the nonlinear system in Equation (10) can be transformed into the following linear system:

(13) $$\{\begin{array}{l} x_{k}=F_{k}x_{k-1}+w_{k-1}\\ z_{k}=H_{z}x_{M_{k}}+v_{k} \end{array}$$

This avoids the linearization process of the measurement function $H_{k}(x_{k})$. Because the system satisfies the linear Gaussian condition of the KF, the mid-state can be updated by the KF. As the optimal linear filter, KF has less information loss and higher filtering accuracy.

**Figure 1:**
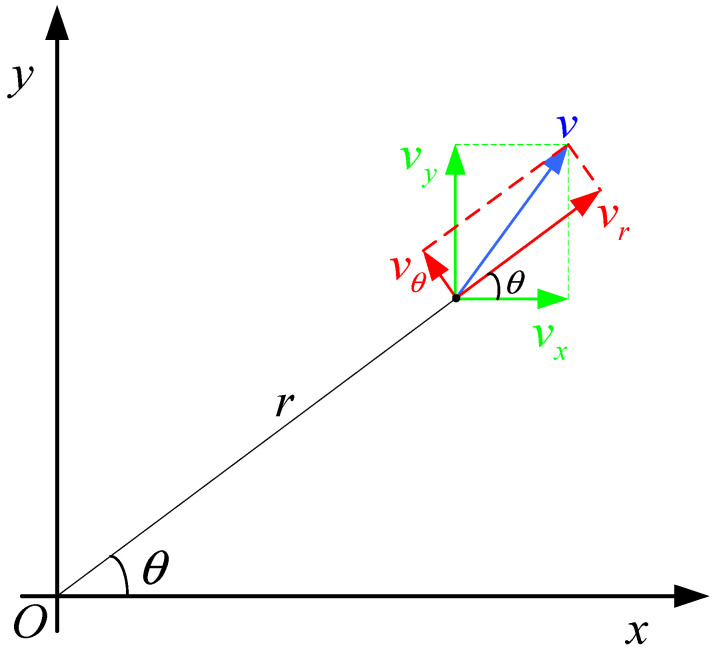
Velocity decomposition.

In the mid-state, ${\left[{\dot{r}}_{k},{\dot{\theta }}_{k}\right]}^{T}$ is not only the first-order state derivative of ${\left[r_{k},\theta_{k}\right]}^{T}$, but also has practical physical significance. ${\dot{r}}_{k}$ and ${\dot{\theta }}_{k}$, respectively, represent the radial velocity $v_{r}$ and angular velocity ω of the target movement in polar coordinates. Therefore, the linear velocity is $v_{\theta }=\omega r$. As shown in Figure 1, the relative radar distance of the target is r, the angle is θ, and the velocity at time k is v. In the cartesian coordinate system, the velocity vector v can be decomposed into x axial velocity $v_{x}$ and y axial velocity $v_{y}$. In the polar coordinate system, the velocity vector v can be decomposed into radial velocity $v_{r}$ and linear velocity $v_{\theta }$. It should be noted that the radial velocity $v_{r}$ only changes the magnitude of the velocity v without changing its direction, while the linear velocity $v_{\theta }$ only changes the direction of the velocity v without changing its magnitude.

The mid-state is introduced into the Kalman filter to form the tracking filter algorithm MSKF, which is suitable for dealing with nonlinear problems. The algorithm steps are given as follows.

**Initialization:** Given the initial state estimate ${\hat{x}}_{0}$ and covariance matrix $P_{0}$, set the time k=1.

**Prediction:** Assume that the input of filter in prediction step is the filtering result ${\hat{x}}_{k}$ and $P_{k}$ at time k, and obtain the state prediction result ${\hat{x}}_{k+1|k}$ and $P_{k+1|k}$ through the following formula:

(14) $${\hat{x}}_{k+1|k}=F_{k}x_{k}$$

(15) $$P_{k+1|k}=F_{k}P_{k}F_{k}^{T}+Q_{k}$$

**State transformation:** The predicted state vector ${\hat{x}}_{k+1|k}$ is converted to the mid-state $x_{M_{k+1|k}}={\left[z_{k+1|k},{\dot{z}}_{k+1|k}\right]}^{T}$, and the predicted covariance $P_{k+1|k}$ in the state space is converted to the covariance $P_{M_{k+1|k}}$ in the measurement space according to the third-order spherical-radial criterion.

According to the geometric relation and velocity decomposition in Figure 1, the transformation relationship from state vector ${\hat{x}}_{k+1|k}={\left[x_{k+1|k},v_{x_{k+1|k}},y_{k+1|k},v_{y_{k+1|k}}\right]}^{T}$ to mid-state vector $x_{M_{k+1|k}}={\left[r_{M_{k+1|k}},{\dot{r}}_{M_{k+1|k}},\theta_{M_{k+1|k}},{\dot{\theta }}_{M_{k+1|k}}\right]}^{T}$ is shown in the following formula:

(16) $$\{\begin{array}{c} r_{M_{k+1|k}}=\sqrt{x_{k+1|k}{}^{2}+y_{k+1|k}{}^{2}}\\ {\dot{r}}_{M_{k+1|k}}=\frac{1}{r}(x_{k+1|k}v_{x_{k+1|k}}+y_{k+1|k}v_{y_{k+1|k}})\\ \theta_{M_{k+1|k}}=\arctan(\frac{y_{k+1|k}}{x_{k+1|k}})\\ {\dot{\theta }}_{M_{k+1|k}}=\frac{1}{r^{2}}(x_{k+1|k}v_{y_{k+1|k}}-y_{k+1|k}v_{x_{k+1|k}}) \end{array}$$

The predicted covariance $P_{k+1|k}$ in the state space was converted to the covariance $P_{M_{k+1|k}}$ in the measurement space according to the third-order spherical-radial criterion:

(1) Obtain 2n sampling points through ${\hat{x}}_{k+1|k}$ and $P_{k+1|k}$:

(17) $$\{\begin{array}{l} w_{i}=w_{i+n_{x}}=\frac{1}{2n_{x}}\\ \zeta_{i}={\hat{x}}_{k+1|k}+\sqrt{n_{x}P_{k+1|k}}e_{i}\\ \zeta_{i+n}={\hat{x}}_{k+1|k}-\sqrt{n_{x}P_{k+1|k}}e_{i} \end{array},\;i=1,2,\cdots ,n_{x}\;$$

where $e_{i}$ represents the unit vector with the i-th element being 1.

(2) The value of the sampling point after conversion is ${\mathbb{C}}_{i}=l(\zeta_{i}),\;i=1,2,\cdots ,2n_{x}$.

(3) Then the covariance $P_{M_{k+1|k}}$ in the intermediate states in the measurement space is:

(18) $$\bar{c}=\sum_{i=1}^{2n}w_{i}{\mathbb{C}}_{i}$$

(19) $$P_{M_{k+1|k}}=\sum_{i=1}^{2n}w_{i}({\mathbb{C}}_{i}-\bar{c}){\left({\mathbb{C}}_{i}-\bar{c}\right)}^{T}$$

The state transformation represents the target state transformation relationship under different coordinates. This module can be quickly replaced according to the actual situation without modifying the filter prediction and update module. This also improves the practical engineering applicability of the MSKF.

**Update:** The state estimation results $x_{M_{k+1}}$ and $P_{M_{k+1}}$ in the measurement space are obtained from the mid-states $x_{M_{k+1|k}}$ and their covariance $P_{M_{k+1|k}}$.

(20) $$S_{M_{k+1}}=H_{z}P_{M_{k+1|k}}H_{z}^{T}+R_{k+1}$$

(21) $$K_{M_{k+1}}=P_{M_{k+1|k}}H_{z}S_{M_{k+1|k}}^{-1}$$

(22) $$x_{M_{k+1}}=x_{M_{k+1|k}}+K_{M_{k+1}}(z_{k+1}-H_{z}x_{M_{k+1|k}})$$

(23) $$P_{M_{k+1}}=(I_{2m}-K_{M_{k+1}}H_{z})P_{M_{k+1|k}}$$

where $H_{z}=[\begin{array}{cc} I_{m} & 0_{m\times m} \end{array}]$ is the observation matrix of the mid-state vector.

**Extraction of state estimation:** Obtain the state estimation ${\hat{x}}_{k+1}$ and $P_{k+1}$ at time k from the filtering results $x_{M_{k+1}}$ and $P_{M_{k+1}}$ in measurement space.

This process is the reverse process of state transformation. Therefore, the conversion relationship from the mid-state vector $x_{M_{k+1}}={\left[r_{M_{k+1}},{\dot{r}}_{M_{k+1}},\theta_{M_{k+1}},{\dot{\theta }}_{M_{k+1}}\right]}^{T}$ to the state vector ${\hat{x}}_{k+1}={\left[x_{k+1},v_{x_{k+1}},y_{k+1},v_{y_{k+1}}\right]}^{T}$ is

(24) $$\{\begin{array}{l} x_{k+1}=r_{M_{k+1}}\cos\theta_{M_{k+1}}\\ v_{x_{k+1}}=v_{r_{M_{k+1}}}\cos\theta_{M_{k+1}}-v_{\theta_{M_{k+1}}}\sin\theta_{M_{k+1}}={\dot{r}}_{M_{k+1}}\cos\theta_{M_{k+1}}-r_{M_{k+1}}{\dot{\theta }}_{M_{k+1}}\sin\theta_{M_{k+1}}\\ y_{k+1}=r_{M_{k+1}}\sin\theta_{M_{k+1}}\\ v_{y_{k+1}}=v_{r_{M_{k+1}}}\sin\theta_{M_{k+1}}+v_{\theta_{M_{k+1}}}\cos\theta_{M_{k+1}}={\dot{r}}_{M_{k+1}}\sin\theta_{M_{k+1}}+r_{M_{k+1}}{\dot{\theta }}_{M_{k+1}}\cos\theta_{M_{k+1}} \end{array}$$

At the same time, $P_{k+1}$ is obtained from $P_{M_{k+1}}$ according to the third-order spherical-radial criterion.

**Figure 2:**
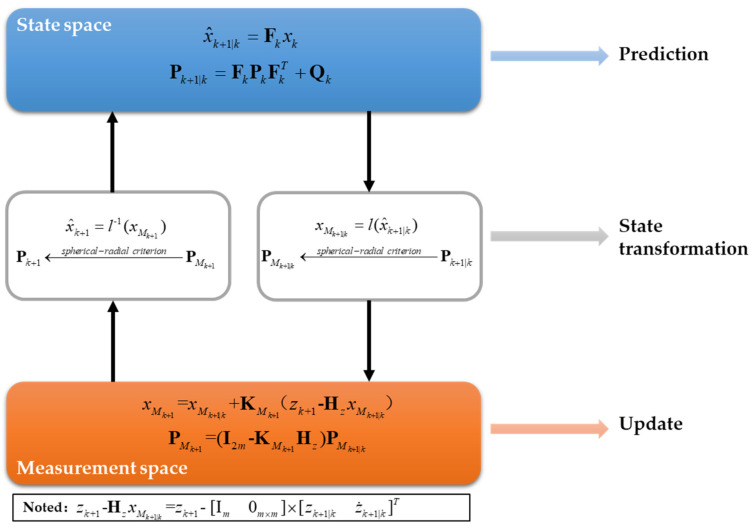
The algorithm flow of the MSKF.

The algorithm flow of the estimator is shown in Figure 2.

## 4. Nonlinear Filter Performance Evaluation

In order to better verify the performance of nonlinear filters, it is necessary to analyze and evaluate the filtering accuracy, credibility, stability, and other aspects of the filter which largely depend on reasonable evaluation metrics. In addition to the common evaluation metrics of state accuracy, such as root mean square error, this paper also deduces and gives the Cramér-Rao Low Bound iteration formula for nonlinear systems and analyzes the consistency of a filter by using a normalized error square.

### 4.1. Root Mean Square Error

Root Mean Square Error (RMSE) is widely used in performance comparison and evaluation of nonlinear filtering algorithms. It is based on the estimation error set obtained by the Monte Carlo simulation to evaluate the accuracy of the algorithm [18], defined as follows:

(25) $$RMSE_{x}=\sqrt{\frac{1}{N_{m}}\sum_{l=1}^{N_{m}}{\left({\hat{x}}_{k|k}^{l}-x_{k}\right)}^{2}}$$

where $N_{m}$ is the Monte Carlo simulation times, ${\hat{x}}_{k|k}^{l}$ represents the estimation result of the target motion state at time k in the l-th Monte Carlo simulation, and $x_{k}$ is the actual state of the target. Although this metric has a definite physical meaning, it is easily dominated by considerable error values.

### 4.2. Cramér-Rao Low Bound for Nonlinear Systems

For nonlinear systems, the optimal Bayesian nonlinear filter is impossible [19], so there are many approximate suboptimal algorithms. They have a theoretical optimal performance lower bound called Cramér-Rao Low Bound (CRLB). It is not only a benchmark for performance analysis, but also can be used to design the parameters of a suboptimal algorithm. It has important theoretical and application value.

Literature [20,21] provides the CRLB iterative formula for discrete nonlinear systems, but it only provides the definition containing the expected operation without the final equations. Therefore, this paper deduces the CRLB for nonlinearity systems with Gaussian white noise.

For the nonlinear system in Equation (1), the unbiased estimate of the state $x_{k}$ is ${\hat{x}}_{k}$ and its covariance matrix is $P_{k}$. The matrix should satisfy:

(26) $$P_{k}=E[({\hat{x}}_{k}-x_{k}){\left({\hat{x}}_{k}-x_{k}\right)}^{T}]\geq J_{k}^{-1}$$

where $J_{k}=E[-\frac{\partial^{2}\ln p(x_{k},z_{k})}{\partial x_{k}^{2}}]$ known as the information matrix, $J_{k}^{-1}$ is the CRLB at time k. The iteration formula is

(27) $$J_{k+1}=D_{k}^{22}-D_{k}^{21}{\left(J_{k}+D_{k}^{11}\right)}^{-1}D_{k}^{12}$$

Among them,

(28) $$\begin{array}{l} D_{k}^{11}=E[-\frac{\partial^{2}\ln p(x_{k+1}|x_{k})}{\partial x_{k}\partial x_{k}}]\\ D_{k}^{12}=E[-\frac{\partial^{2}\ln p(x_{k+1}|x_{k})}{\partial x_{k}\partial x_{k+1}}]\\ D_{k}^{21}=E[-\frac{\partial^{2}\ln p(x_{k+1}|x_{k})}{\partial x_{k+1}\partial x_{k}}]={\left[D_{k}^{12}\right]}^{T}\\ D_{k}^{22}=E[-\frac{\partial^{2}\ln p(x_{k+1}|x_{k})}{\partial x_{k+1}\partial x_{k+1}}]+E[-\frac{\partial^{2}\ln p(z_{k+1}|x_{k+1})}{\partial x_{k+1}\partial x_{k+1}}] \end{array}$$

If both the system noise $v_{k}$ and the measured noise $w_{k}$ are zero-mean Gaussian white noise and their covariance matrices are $Q_{k}$ and $R_{k+1}$, respectively, then it can be obtained from the Formula (28):

(29) $$\frac{\partial \ln p(x_{k+1}|x_{k})}{\partial x_{k}}=\frac{\partial f_{k}^{T}(x_{k})}{\partial x_{k}}Q_{k}^{-1}[x_{k+1}-f_{k}(x_{k})]$$

(30) $$D_{k}^{11}=E\{\frac{\partial f_{k}^{T}(x_{k})}{\partial x_{k}}Q_{k}^{-1}[x_{k+1}-f_{k}(x_{k})]{\left[x_{k+1}-f_{k}(x_{k})\right]}^{T}{\left(Q_{k}^{-1}\right)}^{T}{\left[\frac{\partial f_{k}^{T}(x_{k})}{\partial x_{k}}\right]}^{T}\}=E[{\tilde{F}}_{k}^{T}Q_{k}^{-1}{\tilde{F}}_{k}]$$

(31) $$D_{k}^{12}=-E[{\tilde{F}}_{k}^{T}]Q_{k}^{-1}={\left[D_{k}^{21}\right]}^{T}$$

(32) $$D_{k}^{22}=Q_{k}^{-1}+E[{\tilde{H}}_{k+1}^{T}R_{k+1}^{-1}{\tilde{H}}_{k+1}]$$

Defining ${\tilde{F}}_{k}=\frac{\partial f_{k}(x_{k})}{\partial x_{k}}$ and ${\tilde{H}}_{k+1}=\frac{\partial h_{k+1}(x_{k+1})}{\partial x_{k+1}}$. The initial information matrix $J_{0}$ is the inverse of the initial state covariance matrix $P_{0|0}^{}$:

(33) $$J_{0}=P_{0|0}^{-1}$$

Through the above derivation process, the CRLB provides the lower bound of the mean square error of unbiased estimation and gives the mean square error of an ideal nonlinear filter. In this paper, the RMSE curves of all filters are compared with the CRLB curves of the nonlinear model. The degree of approaching the CRLB curve reflects the accuracy and performance of the corresponding algorithm.

### 4.3. Normalized Estimation Error Squared

Normalized Estimation Error Squared (NEES) gives a more accurate quantitative evaluation of the consistency of the filtering algorithm. Literature [22] gives a relatively standard definition: for the confidence level α(0≤α≤1), when the difference between the real error information and the error information calculated by the filter is not statistically significant, the filter can be considered to be consistent at the level α. The definition is as follows:

(34) $$\varepsilon_{k}=\mathrm{NEES}={\left(x_{k}-{\hat{x}}_{k|k}\right)}^{T}P_{k|k}^{-1}(x_{k}-{\hat{x}}_{k|k})$$

When $P_{k|k}$ is equal to the actual mean square error matrix, $\varepsilon_{k}$ follows the distribution $\chi^{2}$ with degree of freedom $n_{x}$, and $n_{x}$ is the state dimension. In this case, the consistency of state estimation is transformed into a $\chi^{2}$ test problem. Most of the existing filter performance evaluation is based on the Monte Carlo simulation and the Arithmetic-mean Normalized Estimation Error Square (ANEES) using $N_{m}$ simulation results defined as follows:

(35) ζk=ANEES=1Nm∑i=1Nmεkinx=1Nmnx∑i=1Nmεki

## 5. Simulation

### 5.1. Problem Analysis

When the wide-band radar tracks a very long-range target, a typical problem caused by the measurement nonlinearity is the “Contact Lens” problem. It is directly related to the filter update process and is the leading cause of filter inconsistency and divergence.

**Figure 3:**
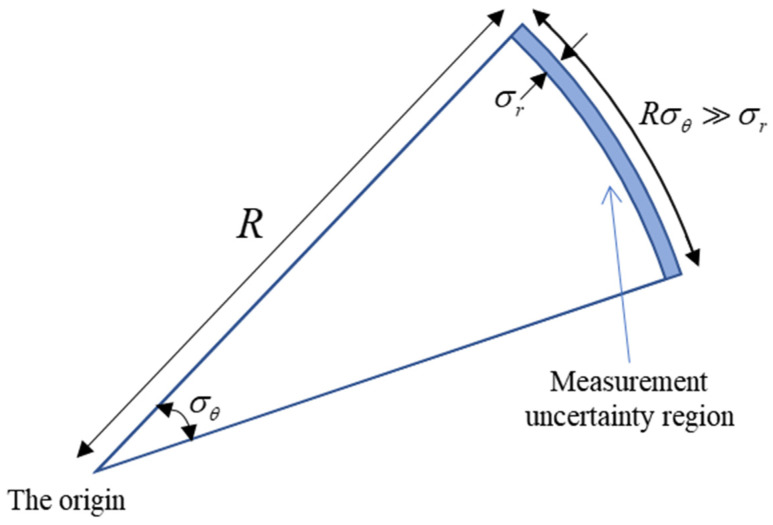
The CL Problem of Nonlinear Measurement.

**Figure 4:**
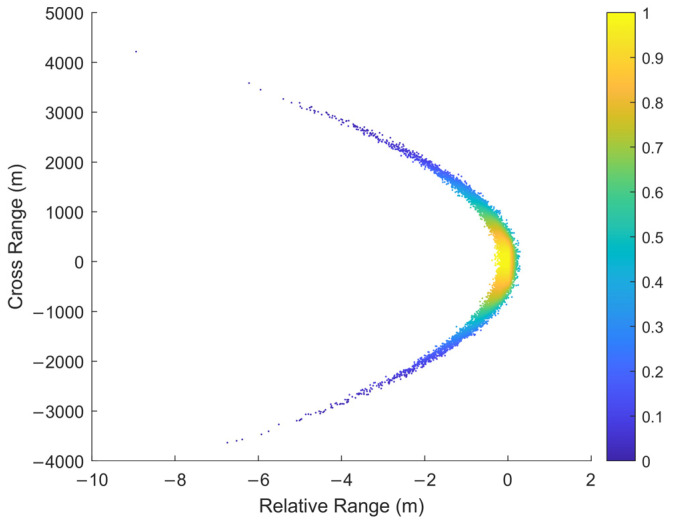
The non-Gaussian CL error distribution.

The wide-band radar has high range accuracy and low angle accuracy when tracking very long-range targets. The uncertainty region of measurement will present a severe non-Gaussian distribution when the state in the Cartesian coordinate system is updated with nonlinear measurements from different coordinate systems. The uncertainty region presents a curved shape, similar to a banana shape in two dimensions and a contact lens shape in three dimensions. Therefore, this distribution is called the “Contact Lens” distribution and this kind of nonlinear problem is called the CL problem [23]. Figure 3 describes the reason for the uncertainty region of measurement from a geometric point of view. In the case of constant range accuracy, the range of cross-angle error $R\sigma_{\theta }^{}$ increases with the radar detection range. As it is far greater than the range error $\sigma_{r}^{}$, the uncertainty region of measurement gradually presents a curve. It should be noticed that in order to describe the distribution characteristics, the curve segment of constant thickness is used in Figure 3 to emphasize the overall curvature of the uncertainty region. However, the curvature of the actual space varies from place to place. The curvature is related to the probability of the measurement distribution at that point in the space. In Figure 4, the intensity of the color is proportional to the probability of the measurement distribution in the space.

**Figure 5:**
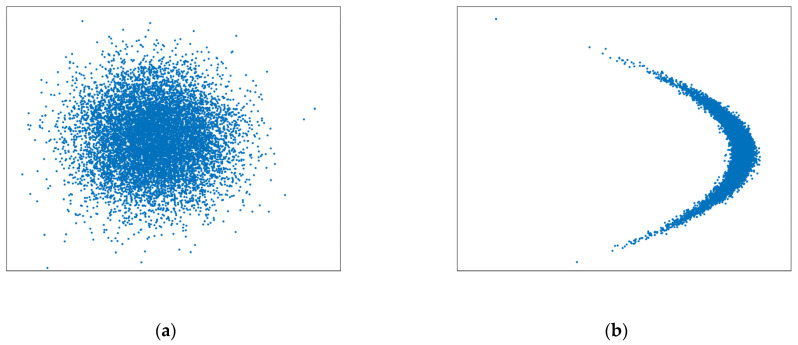
Measurements Distribution: (**a**) $\beta =0.02,\;CL(x;200\;\mathrm{km},{\left(10\;m\right)}^{2},{\left(0.001\;\mathrm{rad}\right)}^{2})$ and (**b**) $\beta =2,\;CL(x;200\;\mathrm{km},{\left(0.1\;m\right)}^{2},{\left(0.001\;\mathrm{rad}\right)}^{2})$.

Bar-shalom uses a parametric expression to describe the uncertainty region $CL(x;r,\sigma_{r}^{2},\sigma_{\theta }^{2})$, but he did not give the specific expression of the distribution. The bias significance metric is put forward to measure the degree of its distortion from a Gaussian distribution [24]:

(36) $$\beta =\frac{r\delta_{\theta }^{2}}{2\delta_{r}}$$

where r is the distance from the target to the radar, $\delta_{\theta }^{2}$ is the radar angle variance, and $\delta_{r}^{2}$ is the radar range variance. Figure 5 shows the measurement distribution characteristics corresponding to different values of β when the target is 500 km away from the radar. When β is small, the measurement distribution shows a typical Gaussian distribution and the traditional filter can track targets well. However, as the value increases, the nonlinear degree of measurement gradually increases. At this time, the nonlinear filters EKF and UKF will have consistency problems, resulting in the decrease of filtering accuracy and even the problem of filter divergence. This problem can be explained by theoretical analysis.

The EKF needs to linearize the measurement equation in Equation (10). Assume (x,y) is the position coordinate of the target relative to the Cartesian coordinate system with the radar as the origin. The vector $\left(x_{0},y_{0}\right)$ is an initial unbiased estimate of (x,y) with the covariance matrix $P_{0}$. The range and angle observation model of the radar is $r={\left(x^{2}+y^{2}\right)}^{1/2}+v_{r}$, $\theta =\arctan(y/x)+v_{\theta }$. Updating $\left(x_{0},y_{0}\right)$ with the measurement r and θ through the EKF, obtains a new estimate $\left(x_{e},y_{e}\right)$ with a covariance matrix $P_{e}$. The EKF valuation equation is then expressed as

(37) $$\left[\begin{array}{c} x_{e}\\ y_{e} \end{array}\right]=\left[\begin{array}{c} x_{0}\\ y_{0} \end{array}\right]+P_{e}H_{0}^{T}R^{-1}\left[\begin{array}{c} r-r_{0}\\ \theta -\theta_{0} \end{array}\right]$$

where

(38) $$P_{e}^{-1}=P_{0}^{-1}+H_{0}^{T}R^{-1}H_{0}$$

(39) $$r_{0}=(\begin{array}{c} {x_{0}^{2}+y_{0}^{2})}^{1/2} \end{array}$$

(40) $$\theta_{0}=\arctan\left(y_{0}/x_{0}\right)$$

(41) $$H_{0}=\left[\begin{array}{cc} \cos\theta_{0} & \sin\theta_{0}\\ -\sin\theta_{0}/r_{0} & \cos0_{0}/r_{0} \end{array}\right]$$

(42) $$R=\left[\begin{array}{cc} \sigma_{r}^{2} & 0\\ 0 & \sigma_{\theta }{}^{2} \end{array}\right]$$

Carrying out the matrix multiplication operation of Formula (37), we can get

(43) $$\left[\begin{array}{c} x_{e}\\ y_{e} \end{array}\right]=\left[\begin{array}{c} x_{0}\\ y_{0} \end{array}\right]+P_{e}\left[\begin{array}{c} \cos\theta_{0}\\ \sin\theta_{0} \end{array}\right][(r-r_{0})/\sigma_{r}^{2}]+P_{e}\left[\begin{array}{c} -\sin\theta_{0}\\ \cos\theta_{0} \end{array}\right][(\theta -\theta_{0})/r_{0}\sigma_{\theta }^{2}]$$

For high-resolution wide-band radar, $P_{0}^{-1}\ll R^{-1}$, so $P_{0}^{-1}$ can be ignored, then

(44) $$P_{e}^{}\approx H_{0}^{-1}R{\left(H_{0}^{T}\right)}^{-1}$$

Thus,

(45) $$\left[\begin{array}{c} x_{e}\\ y_{e} \end{array}\right]\approx \left[\begin{array}{c} x_{0}\\ y_{0} \end{array}\right]+\left[\begin{array}{c} \cos\theta_{0}\\ \sin\theta_{0} \end{array}\right](r-r_{0})+\left[\begin{array}{c} -\sin\theta_{0}\\ \cos\theta_{0} \end{array}\right]r_{0}(\theta -\theta_{0})$$

From the above formula,

(46) $$x_{e}=r\cos\theta_{0}-r_{0}\left(\theta -\theta_{0}\right)\sin\theta_{0}$$

(47) $$y_{e}=r\sin\theta_{0}+r_{0}\left(\theta -\theta_{0}\right)\cos\theta_{0}$$

Therefore, the following formula can be obtained:

(48) $$r_{e}={\left(x_{e}^{2}+y_{e}^{2}\right)}^{1/2}$$

(49) $$\theta_{e}=\arctan\left(y_{e}/x_{e}\right)$$

Assuming that $(r-r_{0})/r$ can be ignored, then the estimation error caused by the linearization of the measurement equation in EKF is

(50) $$r_{e}-r\approx r{\left[1+{\left(\theta -\theta_{0}\right)}^{2}\right]}^{1/2}-r$$

(51) $$\theta_{e}-\theta \approx -(\theta -\theta_{0})+\arctan(\theta -\theta_{0})$$

The above formula shows that when the target is far away from the radar, the estimation error may exceed the distance measurement error $\sigma_{r}^{}$, so the solution of the EKF will tend to diverge.

**Figure 6:**
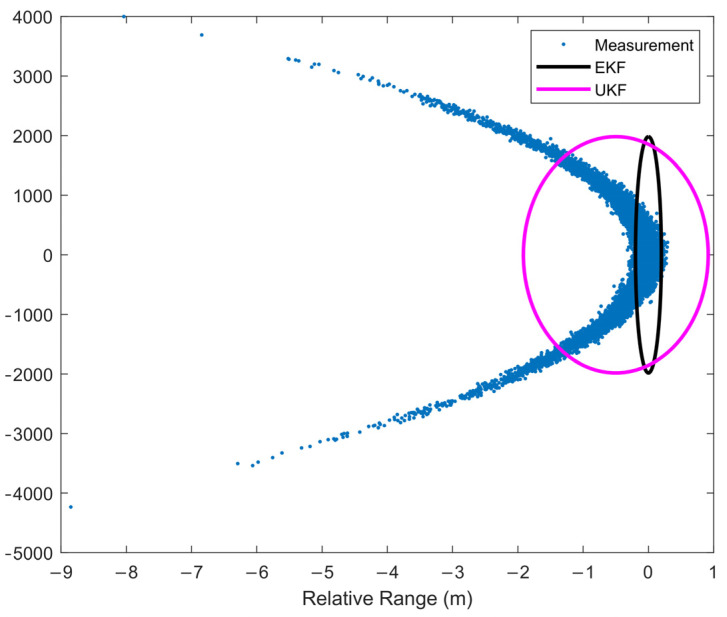
Measurement Distribution and the filter estimation error ellipse.

The UKF is a numerical method to calculate the statistical characteristics of random variables after nonlinear transformation. Its essence is to use multiple deterministic sampling points to approximate the Gaussian probability density function. However, in the CL problem, the probability distribution of measurement in the Cartesian coordinate system obviously cannot be approximated simply by Gaussian distribution, so the filtering accuracy of the UKF is poor. Figure 6 shows the error ellipse of the EKF and UKF estimation results which shows that the Gaussian distribution estimated by the two filters cannot cover the measurement distribution well, so the performance of the two filters is degraded or even divergent.

MSKF uses ${\left[x_{k},{\dot{x}}_{k},y_{k},{\dot{y}}_{k}\right]}^{T}$ as the state vector as the prediction in Cartesian coordinates. The mid-state $\left[r,\dot{r},\theta ,\dot{\theta }\right]$ is selected to make sure the update process is carried out in the measurement coordinates when updating. In the measurement space, the measurement distribution conforms to the Gaussian distribution assumption of the Kalman filter which ensures the consistency of the filter.

### 5.2. Simulations Results

The following two very long-range target tracking scenarios are designed for different radar performances:

(1)
$r=2700\;\mathrm{km},\delta_{r}=10\;m,\delta_{\theta }=0.3^{\circ },\beta =3.7;$

(2)
$r=2700\;\mathrm{km},\delta_{r}=0.1\;m,\delta_{\theta }=0.3^{\circ },\beta =371$

**Figure 7:**
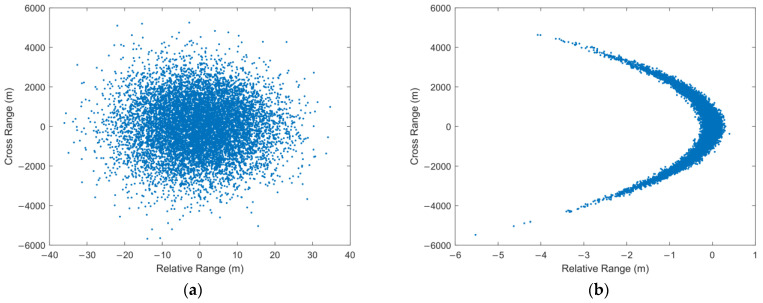
Measurements Distribution: (**a**) $\beta =3.7,\;CL(x;2700\;\mathrm{km},{\left(10\;m\right)}^{2},{\left(0.3^{\circ }\right)}^{2})$ and (**b**) $\beta =371,\;CL(x;2700\;\mathrm{km},{\left(0.1\;m\right)}^{2},{\left(0.3^{\circ }\right)}^{2})$.

Figure 7 shows the measurement distribution of two simulation scenarios. Simulation scenario 1 uses narrowband radar to track very long-range targets. This is set according to the situation of missile target tracking by narrowband radar in engineering. The measurement distribution is Gaussian distribution. Simulation scenario 2 uses wide-band radar to track very long-range targets. In scenario 2, the typical CL problem occurs.

The experiment uses 100 Monte Carlo simulations, the initial target position is $x_{0}=1050\;\mathrm{km}$, $y_{0}=2500\;\mathrm{km}$, and the initial velocity is $v_{x}=200\;m/s$,$v_{y}=300\;m/s$. The traditional nonlinear filters EKF, UKF, and CKF are used for performance comparison simulation. The sampling time interval of the four filters is T=0.1 s and the tracking time is 80 s. RMSE, CRLB theory, and consistency metric ANEES are used to test the filtering performance. The convergence speed of the filter is greatly related to the initial state, so the two-point starting method is adopted. The initial state and covariance are obtained by differentiating the two measurements at the first time and the second time.

Table 1 lists the range and velocity estimation RMSE of different filters in the tracking scenario (1) when the measurement uncertainty region is Gaussian distribution. In order to compare the computational complexity, the table also lists the Average State Estimation Time Consumption (ASETC) of different filters. It can be seen from Table 1 that the range measurement accuracy of the MSKF is similar to that of the UKF and CKF, which shows that the MSKF is also suitable for nonlinear target tracking in general. The angle measurement accuracy of the CKF and MSKF is higher than the EKF and UKF. The time cost of the MSKF is slightly higher than that of the other filters, but it increases the accuracy of radar angle measurement. In Table 2, the MSKF improves the angle measurement accuracy and achieves accurate tracking of a very long-range target compared with other filters. In summary, the MSKF can be used in various situations and, for special nonlinear scenarios, it can also effectively improve radar tracking accuracy.

**Figure 8:**
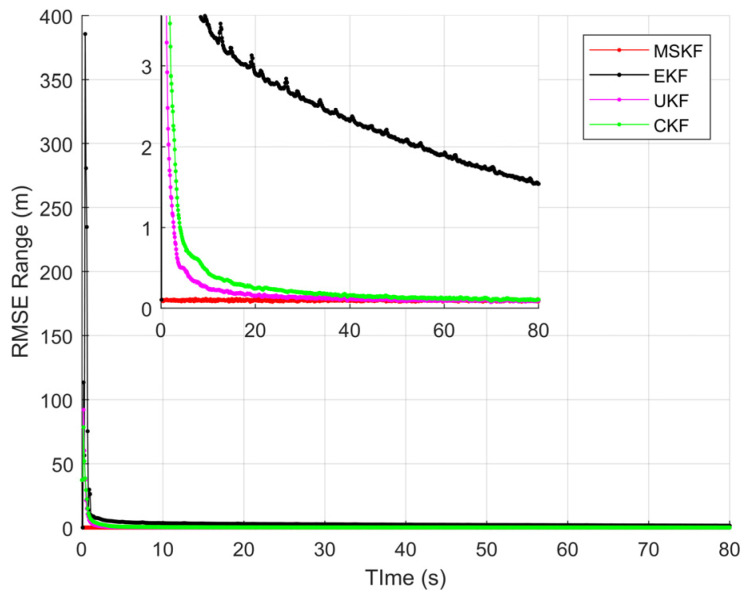
Range estimation accuracy of CL distribution.

**Figure 9:**
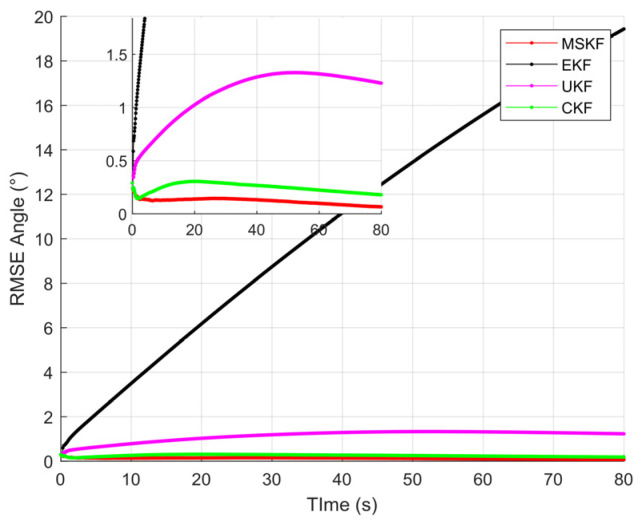
Angle estimation accuracy of CL distribution.

**Figure 10:**
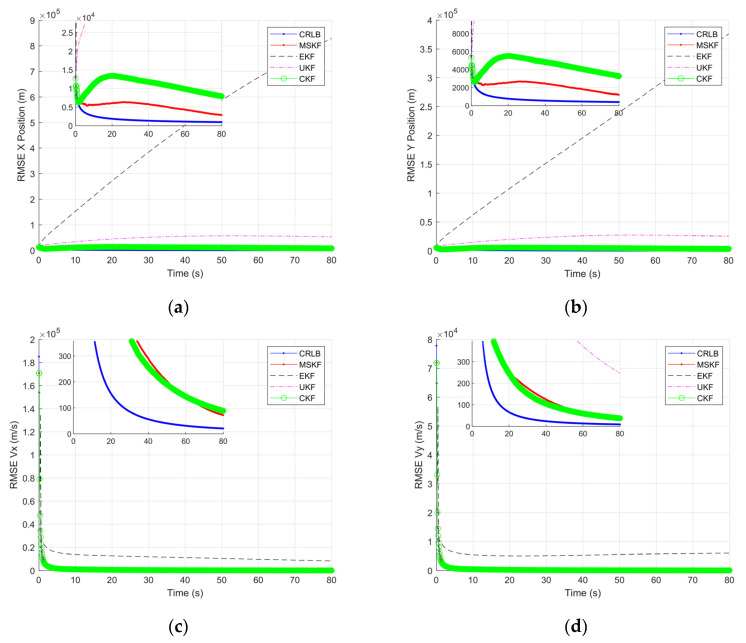
Comparison of state component RMSE and CRLB of CL distribution: (**a**) RMSE in X position; (**b**) RMSE in Y position; (**c**) RMSE in X speed; and (**d**) RMSE in Y speed.

**Figure 11:**
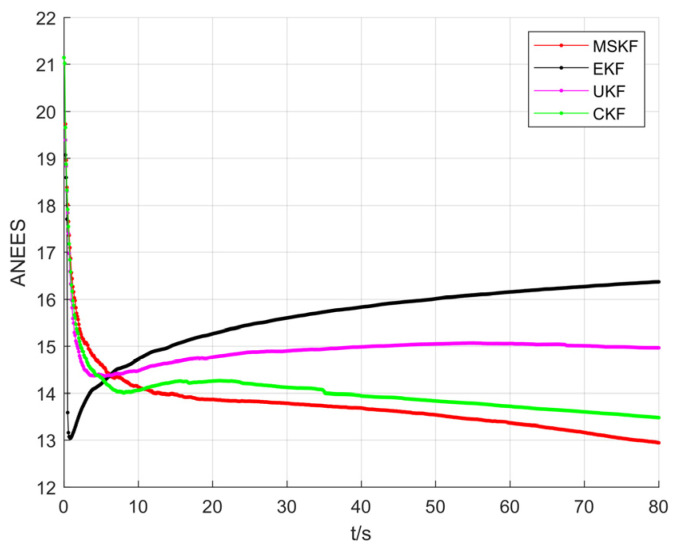
Comparison of ANEES of CL distribution.

Figure 8, Figure 9, Figure 10 and Figure 11 shows the simulation results of tracking very long-range targets with filters in scenario (2). At the time β=371, the uncertainty region of measurement is CL distribution. It can be seen from Figure 8 and Figure 9 that for very long-range targets, the measurement uncertainty region presents a severe non-Gaussian shape and the three filters except EKF can accurately track the target. In order to achieve the linear approximation of the system model, the EKF ignores the higher-order terms of Taylor’s expansion. Therefore, the EKF causes a significant error in the estimated state posterior distribution due to the truncation error. The UKF and CKF design a small number of deterministic sampling points and approximate the posterior probability of the state by calculating the statistical characteristics of these sampling points after passing through the nonlinear system so the target state is estimated accurately. For the tracking scenario (2), the angle estimation accuracy of the CKF is higher than that of the UKF. As shown in Table 2, the MSKF proposed in this paper has better filtering performance and higher target tracking accuracy than the CKF when the uncertainty region of measurement is CL distribution. Meanwhile, since the MSKF performs linear updates in the measurement space and is more affected by measures, the convergence speed of the MSKF is faster under the same two-point starting mode.

The state component estimation RMSE and theoretical performance lower bound CRLB is given in Figure 10. Due to the non-Gaussian measurement distribution, the initial RMSE of the four filters and CRLB are all large. As the iteration progresses, the UKF, CKF, and MSKF proposed in this paper gradually converge to steady-state, while the range accuracy of the EKF gradually diverges. The steady-state value indicates the approximation performance of the algorithm to nonlinear filtering. The convergence rate of RMSE represents the speed at which the algorithm finds the center of the non-Gaussian distribution. Figure 10a,b clearly show that the CKF and MSKF gradually converge to CRLB, and the MSKF has a faster convergence rate, a smaller steady-state value, and is closer to the theoretical boundary.

Figure 11 shows the ANEES distribution of the four filters. Since the consistency metric ANEES measures the difference between the real error and the estimated covariance matrix, the smaller the value, the better the consistency and the higher the robustness. It can be seen that the ANEES of EKF increases gradually, which means that the covariance matrix of the state vector increases gradually in the estimation process, eventually leading to the divergence. The ANEES of UKF gradually converges to a constant value, but its robustness is poor which leads to a significant estimation error. However, the ANEES of the CKF and MSKF gradually converge to achieve high-precision and stable target tracking. The MSKF has better state covariance estimation and higher robustness.

## 6. Conclusions

A typical nonlinear CL problem occurs when high-resolution wide-band radar tracks very long-range targets. To solve this problem, a novel mid-state Kalman filter is proposed in this paper. The MSKF uses the observed state and its first state derivative as the mid-state vector to transform the update process of the filter into the measurement space and rederives the update equation of the Kalman filter. The state transition module can be replaced according to the actual situation without modifying the filter prediction and update module. This improves the practical engineering applicability of the MSKF. When the CL problem occurs, the MSKF has the characteristics of high state estimation accuracy and fast filter convergence compared with other filters. This will significantly help the wide-band radar system to form the target trajectory quickly and accurately.

## Figures and Tables

**Table 1:** Different filtering accuracy and time consumption in scenario (1).

Filter	Range RMSE/m	Angle RMSE/°	ASETC/s
EKF	2.487	0.2224	0.0295
UKF	2.506	0.0986	0.0450
CKF	2.502	0.0266	0.0426
MSKF	2.598	0.0208	0.0691

**Table 2:** Different filtering accuracy and time consumption in scenario (2).

Filter	Range RMSE/m	Angle RMSE/°	ASETC/s
EKF	1.6700	17.7700	0.03155
UKF	0.0956	1.2730	0.04779
CKF	0.1139	0.1977	0.04578
MSKF	0.0926	0.0781	0.07162

## Data Availability

The data presented in this study are available on request from the corresponding author.
